# Supplementary material for: Single-Step IGHV Next-Generation Sequencing Detects Clonality and Somatic Hypermutation in Lymphoid Malignancies: A Phase III Diagnostic Accuracy Study
Source: Cancers (Basel). 2023 Sep 19;15(18):4624. doi: 10.3390/cancers15184624 (PMC10526376; doi:10.3390/cancers15184624)
Supplement: Supplementary file 1 [file cancers-15-04624-s001.zip › cancers-2554445-supplementary.pdf]

Supplementary Table S1: STARD requirements

Section and Topic	Item #		On page #
TITLE/ABSTRACT/ KEYWORDS	1	Identify the article as a study of diagnostic accuracy (recommend MeSH heading 'sensitivity and specificity').	1,5
INTRODUCTION	2	State the research questions or study aims, such as estimating diagnostic accuracy or comparing accuracy between tests or across participant groups.	4-6
METHODS			
Participants	3	Describe the study population: The inclusion and exclusion criteria, setting and locations where the data were collected.	6
	4	Describe participant recruitment: Was recruitment based on presenting symptoms, results from previous tests, or the fact that the participants had received the index tests or the reference standard?	Consecutive patients who received the index test
	5	Describe participant sampling: Was the study population a consecutive series of participants defined by the selection criteria in items 3 and 4? If not, specify how participants were further selected.	Consecutive cases
	6	Describe data collection: Was data collection planned before the index test and reference standard were performed (prospective study) or after (retrospective study)?	Restrospective study
Test methods	7	Describe the reference standard and its rationale.	4-5
	8	Describe technical specifications of material and methods involved including how and when measurements were taken, and/or cite references for index tests and reference standard.	6-11
	9	Describe definition of and rationale for the units, cutoffs and/or categories of the results of the index tests and the reference standard.	11-12
	10	Describe the number, training and expertise of the persons executing and reading the index tests and the reference standard.	/
	11	Describe whether or not the readers of the index tests and reference standard were blind (masked) to the results of the other test and describe any other clinical information available to the readers.	/
Statistical methods	12	Describe methods for calculating or comparing measures of diagnostic accuracy, and the statistical methods used to quantify uncertainty (e.g. 95% confidence intervals).	11-13

	13	Describe methods for calculating test reproducibility, if done.	/
RESULTS			
Participants	14	Report when study was done, including beginning and ending dates of recruitment.	/
	15	Report clinical and demographic characteristics of the study population (e.g. age, sex, spectrum of presenting symptoms, comorbidity, current treatments, recruitment centers).	/
	16	Report the number of participants satisfying the criteria for inclusion that did or did not undergo the index tests and/or the reference standard; describe why participants failed to receive either test (a flow diagram is strongly recommended).	11-14
Test results	17	Report time interval from the index tests to the reference standard, and any treatment administered between.	/
	18	Report distribution of severity of disease (define criteria) in those with the target condition; other diagnoses in participants without the target condition.	/
	19	Report a cross tabulation of the results of the index tests (including indeterminate and missing results) by the results of the reference standard; for continuous results, the distribution of the test results by the results of the reference standard.	21-23 table 1 - 25-28 table 4
	20	Report any adverse events from performing the index tests or the reference standard.	/
Estimates	21	Report estimates of diagnostic accuracy and measures of statistical uncertainty (e.g. 95% confidence intervals).	13
	22	Report how indeterminate results, missing responses and outliers of the index tests were handled.	14
	23	Report estimates of variability of diagnostic accuracy between subgroups of participants, readers or centers, if done.	/
	24	Report estimates of test reproducibility, if done.	/
DISCUSSION	25	Discuss the clinical applicability of the study findings.	15-19

Supplementary Table S2: QUADAS 2 requirements

Phase 1: State the review question:	
Patients (setting, intended use of index test, presentation, prior testing):	.68 samples of B-lymphoproliferative disorders (51 chronic lymphocytic leukemia , 8 Follicular Lymphoma , 3 mantle cell lymphoma and 6 reactive lymphoid hyperplasia-) obtained from peripheral blood or formalin-fixed paraffin-embedded tissue were included. the index tests were applied to verify the diagnostic accuracy of NGS technologies (LymphoTrackTM IGH Somatic Hypermutation Assay) to evaluate clonality and hypermutation. prior testing was represented by the reference standard
Index test(s):	1. IGH Clonal Analysis: NGS technologies (LymphoTrackTM IGH Somatic Hypermutation Assay)vs PCR BIOMED2
	2. IGH hypermutation analysis :NGS technologies (LymphoTrackTM IGH Somatic Hypermutation Assay)vs PCR BIOMED2 followed by Sanger Sequencing
Reference standard and target condition:	Reference standard was performed by Polymerase Chain Reaction (PCR) assays followed by capillary electrophoresis and/or Sanger sequencing according to the Euroclonality and ERIC guidelines
Phase 2: Draw a flow diagram for the primary study	
**DOMAIN 1: PATIENT SELECTION**	
**A. Risk of Bias**	
Describe methods of patient selection:	
Was a consecutive or random sample of patients enrolled?	Consecutive
Was a case-control design avoided?	Yes
Did the study avoid inappropriate exclusions?	Yes
Could the selection of patients have introduced bias?	risk low
**B. Concerns regarding applicability**	
Describe included patients (prior testing, presentation, intended use of index test and setting):	68 samples of B-lymphoproliferative disorder were analized by NGS and references standard to establish the diagnostic accuracy of NGS technologies in clonality and Hypermutation assessment of IGH. Diagnoses (made with the reference standard) were reviewed by at least two very experienced haematopathologists. The studied samples were collected at diagnosis time. The index test was applied retrospectively to all the cases.
Is there concern that the included patients do not match the review question?	Concern: low
**DOMAIN 2: INDEX TEST(S) IGH Clonal Analysis by NGS vs. PCR**	
**A. Risk of Bias**	
Describe the index test and how it was conducted and interpreted:	we compared the NGS assay to reference standard to verify its diagnostic accuracy concernig clonality assessment. The results were interpretaded acconding to euroclonality guidelines
Were the index test results interpreted without knowledge of the results of the reference standard?	Yes
If a threshold was used, was it pre-specified?	Yes
Could the conduct or interpretation of the index test have introduced bias?	risk low
**B. Concerns regarding applicability**	
Is there concern that the index test, its conduct, or interpretation differ from the review question?	Concern: low
**DOMAIN 2: INDEX TEST(S) 2. IGH hypermutation analysis NGS vs. PCR/ Sanger sequencing**	

**A. Risk of Bias**<br>Describe the index test and how it was conducted and interpreted:<br><br>Were the index test results interpreted without knowledge of the results of the reference standard?<br>If a threshold was used, was it pre-specified?<br>Could the conduct or interpretation of the index test have introduced bias?	we compared the NGS assay to reference standard to verify its diagnostic accuracy concernig Hypermutation assessment of IGH. The results were interpretaded according to euroclonality and ERIC guidelines<br>Yes<br><br>Yes<br>risk low
**B. Concerns regarding applicability**<br>Is there concern that the index test, its conduct, or interpretation differ from the review question?	Concern: low
**DOMAIN 3: REFERENCE STANDARD**	
**A. Risk of Bias**<br>Describe the index test and how it was conducted and interpreted:<br><br>Is the reference standard likely to correctly classify the target condition?<br>Were the reference standard results interpreted without knowledge of the results of the index test?<br>Could the reference standard, its conduct, or its interpretation have introduced bias?	Polymerase Chain Reaction (PCR) assays followed by capillary electrophoresis and/or Sanger sequencing was performed and interpretaded according to the Euroclonality and ERIC guidelines,. These tests use a mixture of consensus primers designed to amplify and to sequence the majority of possible unique V(D)J rearrangements. In this manner, clonal proliferations can be detected with very high sensitivity and specificity<br>Yes<br><br>Yes<br>risk low
**B. Concerns regarding applicability**<br>Is there concern that the target condition as defined by the reference standard does not match the review question?	Concern: low
**DOMAIN 4: FLOW AND TIMING**	
**A. Risk of Bias**<br>Describe any patients who did not receive the index test(s) and/or reference standard or who were excluded from the 2x2 table (refer to flow diagram)<br>Describe the time interval and any interventions between index test(s) and reference standard:<br>Was there an appropriate interval between index test(s) and reference standard?<br>Did all patients receive a reference standard?<br>Did patients receive the same reference standard?<br>Were all patients included in the analysis?<br>Could the patient flow have introduced bias?	All the patients enrolled received both.<br><br>Samples were collected at diagnosis . At that time, the references standard was performed. At the time of the study (2014), index test was performed on the same cases.<br>yes<br><br>yes<br>yes<br>yes<br>risk low

Supplementary Table S3: REMARK requirements

**Guidelines for the REporting of tumor MARKer Studies (REMARK)**	**See page**
Introduction	
1. State the marker examined, the study objectives, and any prespecified hypotheses.	4-6
Materials and Methods	
Patients	
2. Describe the characteristics (eg, disease stage or comorbidities) of the study patients, including their source and inclusion and exclusion criteria.	6
3. Describe treatments received and how chosen (eg, randomized or rule-based).	Not applicable
Specimen characteristics	
4. Describe the type of biological material used (including control samples) and methods of preservation and storage.	6
Assay methods	
5. Specify the assay method used and provide (or reference) a detailed protocol, including specific reagents or kits used, quality control procedures, reproducibility assessments, quantitation methods, and scoring and reporting protocols. Specify whether and how assays were performed blinded to the study end point.	7-11
Study design	
6. State the method of case selection, including whether the study design was prospective or retrospective and whether stratification or matching (eg, by stage of disease or age) was used. Specify the time period from which cases were taken, the end of the follow-up period, and the median follow-up time.	Not applicable
7. Precisely define all clinical end points examined.	Not applicable
8. List all candidate variables initially examined or considered for inclusion in models.	Not applicable
9. Give rationale for sample size; if the study was designed to detect a specified effect size, give the target power and effect size.	Not applicable
Statistical analysis methods	
10. Specify all statistical methods, including details of any variable selection procedures and other model-building issues, how model assumptions were verified, and how missing data were handled.	11
11. Clarify how marker values were handled in the analyses; if relevant, describe methods used for cutpoint determination.	/
Results	
Data	

12. Describe the flow of patients through the study, including the number of patients included in each stage of the analysis (a diagram may be helpful) and reasons for dropout. Specifically, both overall and for each subgroup extensively examined report the numbers of patients and the number of events.	Not applicable
13. Report distributions of basic demographic characteristics (at least age and sex), standard (disease-specific) prognostic variables, and tumor marker, including numbers of missing values.	Not applicable
Analysis and presentation	
14. Show the relation of the marker to standard prognostic variables.	Not applicable
15. Present univariate analyses showing the relation between the marker and outcome, with the estimated effect (eg, hazard ratio and survival probability). Preferably provide similar analyses for all other variables being analyzed. For the effect of a tumor marker on a time-to-event outcome, a Kaplan-Meier plot is recommended.	Not applicable
16. For key multivariable analyses, report estimated effects (eg, hazard ratio) with confidence intervals for the marker and, at least for the final model, all other variables in the model.	12
17. Among reported results, provide estimated effects with confidence intervals from an analysis in which the marker and standard prognostic variables are included, regardless of their statistical significance.	Table 3
18. If done, report results of further investigations, such as checking assumptions, sensitivity analyses, and internal validation.	/
Discussion	
19. Interpret the results in the context of the prespecified hypotheses and other relevant studies; include a discussion of limitations of the study.	15-19
20. Discuss implications for future research and clinical value.	15-19
